# Covalent Immobilization of β-Glucosidase into Mesoporous Silica Nanoparticles from Anhydrous Acetone Enhances Its Catalytic Performance

**DOI:** 10.3390/nano10010108

**Published:** 2020-01-05

**Authors:** Filomena Sannino, Aniello Costantini, Francesco Ruffo, Antonio Aronne, Virginia Venezia, Valeria Califano

**Affiliations:** 1 Department of Agricultural Sciences, Università degli Studi di Napoli Federico II, Via Università 100, 80055 Portici (Na), Italy; fsannino@unina.it; 2 Department of Chemical Engineering, Materials and Industrial Production, Università degli Studi di Napoli Federico II, P.le Tecchio 80, 80125 Napoli, Italy; anaronne@unina.it (A.A.); virginia.venezia@libero.it (V.V.); 3 Department of Chemical Science, Università degli Studi di Napoli Federico II, Complesso Universitario di Monte S. Angelo via Cintia, 80126 Napoli, Italy; ruffo@unina.it; 4 Istituto Motori-CNR, via G. Marconi 4, 80125 Napoli, Italy; v.califano@im.cnr.it

**Keywords:** covalent immobilization, β-glucosidase, mesoporous silica nanoparticles

## Abstract

An immobilization protocol of a model enzyme into silica nanoparticles was applied. This protocol exploited the use of the bifunctional molecule triethoxysilylpropylisocyanate (TEPI) for covalent binding through a linker of suitable length. The enzyme β-glucosidase (BG) was anchored onto wrinkled silica nanoparticles (WSNs). BG represents a bottleneck in the conversion of lignocellulosic biomass into biofuels through cellulose hydrolysis and fermentation. The key aspect of the procedure was the use of an organic solvent (anhydrous acetone) in which the enzyme was not soluble. This aimed to restrict its conformational changes and thus preserve its native structure. This approach led to a biocatalyst with improved thermal stability, characterized by high immobilization efficiency and yield. It was found that the apparent ***K_M_*** value was about half of that of the free enzyme. The ***V_max_*** was about the same than that of the free enzyme. The biocatalyst showed a high operational stability, losing only 30% of its activity after seven reuses.

## 1. Introduction

Enzymes are catalysts with excellent properties and high activity, selectivity, and specificity. They can promote complex chemical processes under mild environmental conditions [1,2]. However, they have several features that limit their use for industrial applications, such as inhibition by high concentrations of substrates and products, low stability and reusability, and high cost. The possibility of improving these unsuitable characteristics via the design of simple immobilization protocols is a very important goal. Immobilization techniques have proven to be a powerful tool in improving enzyme properties, if properly designed [3,4,5]. The use of a relatively expensive biocatalyst such as an enzyme requires its recovery and reuse, so that the process can be economically feasible. Simplicity, cost-effectiveness, and stabilization of enzymes are the key requirements for successful industrial application. So far, various carriers and methodologies have been used for enzyme immobilization [3,4,5]. The immobilization of biocatalysts on/in a solid matrix can reduce the production cost by both increasing efficiency and allowing reuse. Moreover, it eases the separation of the products from the reaction mixture. Furthermore, enzyme immobilization often results in the stabilization of enzymes, sometimes increasing or modifying their activity [3].

Among the possible enzymatic immobilization methods, the physical one is the simplest and can be carried out under mild conditions [5]. With this method, the enzyme activity is often preserved, but the immobilized enzyme can have poor operation stability and be subjected to leaching [6]. Enzyme immobilization by covalent binding can anchor the biocatalyst to the support firmly and avoid the shedding and leakage of the enzyme. However, this method may lead to the loss of the enzyme active conformation caused by harsh reaction conditions or by a strong interaction with the support [7].

The aim of this study is the development of an immobilization protocol through covalent binding of a model enzyme into silicate porous nanoparticles using the bifunctional molecule triethoxysilylpropylisocyanate (TEPI). This methodology is based on the use of an organic solvent (anhydrous acetone) in which the enzyme is insoluble so to restrict its conformational freedom and thus preserve its native structure. This should reduce the enzyme inactivation and greatly increase its stability. The novelty of the work is that the whole immobilization procedure is carried out in anhydrous acetone, starting from a suspension of lyophilized β-glucosidase (BG) powders. During the entire process, the lyophilized BG is never in contact with water because the reaction system is kept under dry nitrogen flux. It is demonstrated that solvents that do not dissolve proteins, such as acetone, cause no appreciable denaturation [8]. In fact, enzymes are catalytically active in aprotic organic solvents because they remain trapped in their native conformation [9].

It is well known that change of medium to anhydrous organic solvents can improve enzyme thermal stability [10]. One of the first studies on immobilization from a pure organic solvent (*n*-hexane) concerned lipase [11], whereas other studies investigated the immobilization of enzymes from organic solvents containing small amounts of water (microaqueous organic media). It is claimed that in these kinds of media enzymes become more rigid and then less susceptible to conformational changes [12]. In fact, most enzymes are flexible in water, and covalent immobilization generally leads to their inactivation [13]. Due to their hydrophobic/hydrophilic nature, mostly lipases have been immobilized from organic media [12,14,15], but also other enzymes [12,13]. The performance of the immobilized enzymes resulted in superior activity compared to those immobilized from aqueous media.

In this work, for the first time, the selected enzyme β-glucosidase (BG) was immobilized from an organic solvent. BG plays a key role in the valorization of lignocellulosic biomass for the production of fine chemicals and biofuels (bioethanol) [16]. BG hydrolyzes short chain sugars (i.e., cellobiose) into fermentable glucose molecules. Cellobiose hydrolysis is the rate-limiting factor for the whole process of the enzymatic degradation of cellulose because it acts as inhibitor for cellulolytic enzymes. The urgent need to create new conversion pathways to reduce the dependency on fossil resources requires effective actions to identify innovative and more sustainable bio-based supply chains.

BG has been covalently immobilized on a variety of supports [17,18,19,20,21,22]. Covalent binding on chitosan led to enhanced BG thermal and storage stability [17], but chitosan has limited industrial applications for its very soft texture and density similar to water. Due to their peculiarity, silica nanoparticles represent a broad platform for many applications in different fields [23,24,25,26]. The immobilization of BG onto silica gel [19] and silica nanoparticles [20] led to improved activity. Increase in pH and storage stability was also obtained using macroporous carrier Eupergit C [18]. β-Glucosidase covalently immobilized on nanoparticle carriers has been studied by several authors [20,27,28,29]. However, the use of mesoporous nanoparticles can lead to a number of advantages due to their high surface area and pore size [30]. The porous structure protects the enzyme against the harsh conditions of the reaction environment, such as high temperatures, extreme pH, and the presence of bubbles or inhibitors, creating an ideal micro-environment for the expression of the enzymatic activity [31,32]. Wrinkled silica nanoparticles (WSNs) were chosen as immobilization matrix. It was recently demonstrated that this matrix is a very efficient support for the physical adsorption of BG—its peculiar morphology created a favorable microenvironment for catalysis, reducing diffusion limitations [33,34].

The covalent immobilization protocol led to a biocatalyst with high activity, immobilization yield, thermal stability, and reusability.

## 2. Materials and Methods

For nanoparticle synthesis, tetraethylorthosilicate (TEOS), urea, cetylpyridinium bromide (CPB), cyclohexane, isopropanol, acetone, 37% hydrochloric acid solution, citric acid, and trisodium citrate were used. All chemicals were purchased from Sigma-Aldrich (Milan, Italy). For enzyme immobilization and activity measurements, β-glucosidase from almond (molecular weight 135,000 Da for the dimer), sodium hydroxide, TEPI (95% volume ratio), and glucose oxidase-peroxidase (GOD-POD) assay kit from Sigma-Aldrich (Milan, Italy) were used. The activity of BG, that is, the amount of enzyme that liberates 1 μmol of glucose per minute at pH 5.0 and 37 °C (salicin as substrate) was provided from the producer (≥6 U/mg).

Citrate buffer solution at pH 5.0 was prepared as specified in [35]. A total of 3.9 mg of citric acid was dissolved in 205 mL of bidistilled water, and 8.7 mg of trisodium citrate was dissolved in 295 mL of bidistilled water. The two solutions were then mixed and the volume was brought to 1 L.

### 2.1. Nanoparticle Synthesis

Silica mesoporous nanoparticles with radial wrinkle structure were prepared following the procedure reported in the literature [33]. Briefly, a water/surfactant/oil ternary system composed of cetyltrimethylammonium bromide as surfactant, aqueous urea as water phase, and *n*-butanol/cyclohexane as oil phase was used as template for the synthesis of the wrinkled nanostructures [36]. TEOS was used as silica precursor. The solution was stirred for 30 min at room temperature, then heated up to 70 °C and kept for 16 h in a closed vessel to avoid solvent evaporation. The nanoparticles thus obtained were then washed with ethanol and hydrochloric acid to remove the surfactant.

### 2.2. BG Covalent Immobilization Procedure

Two biocatalysts (BGI2 and BGI9) were prepared by varying the amount of the crosslinking agent, 2 and 9 μL, respectively. The crosslinking agent was TEPI in both cases. These two different volumes were chosen to stoichiometrically react TEPI with some or all the –NH_2_ groups of the protein side chain. The content of –NH_2_ groups of the enzyme was provided by the UniProt database. The molar concentration in the reaction batch were 0.088 mM for BG, 6.2 mM of –NH_2_ groups, and 6.5 or 1.5 mM for TEPI.

The immobilization of BG was carried out in two steps. Step one involved the reaction between 30 mg of enzyme and 2 or 9 μL of TEPI in 5 mL of anhydrous acetone at room temperature for 24 h. Lyophilized BG powders were first suspended in anhydrous acetone under gentle stirring and nitrogen flux, and then TEPI was added. In the second step, 100 mg of nanoparticles dispersed in 2 mL of anhydrous acetone were added to the reaction mixture under N_2_ flux and the reaction was carried out for 7 h at 50 °C under reflux. The products were isolated by centrifugation and washed once with acetone and twice with buffer solution (pH 5.0). The prepared biocatalysts were kept in 2 mL of buffer solution at 4 °C before use.

The yield of immobilization (**IY**) was evaluated from the following equation:

$$\mathrm{IY}\;(\%)=\frac{C_{i}-C_{s}}{C_{i}}\cdot 100$$

where **C_i_** is the protein initial concentration (mg/mL) and **C_s_** is protein concentration measured in the reaction mixture at the end of the immobilization reaction. The Bradford method was used to determine the protein concentration, using bovine serum albumin (BSA) as standard [37]. The immobilization efficiency was evaluated as a relative activity—activity of immobilized enzyme/activity of free enzyme. The enzyme activity is defined as the moles of substrate converted per unit time. The amount of immobilized enzyme was expressed as a percentage ratio in weight—wt of adsorbed protein/wt of support × 100.

### 2.3. BG Physical Immobilization Procedure

For comparison, BG was also immobilized into WSNs by physical adsorption. The adsorption was carried out by mixing a colloidal solution of WSNs of 2 mg/mL to a β-glucosidase 0.03 mM buffer solution at pH 5 and 25 °C. The volume ratio of the two solutions was 0.25. The resulting colloidal solution was gently stirred for 24 h. The resulting nanoparticles were recovered by centrifugation at 11,000 rpm for 10 min and washed twice with the buffer.

### 2.4. Material Characterization

Fourier transform infrared (FT-IR) spectra were acquired by a Nexus FTIR spectrometer equipped with a DTGS KBr (deuterated triglycine sulfate with potassium bromide windows) detector. The spectra were acquired in the range 4000−400 cm^−1^ with a spectral resolution of 2 cm^−1^. Each spectrum represents an average of 32 scans, corrected for the spectrum of the blank KBr pellet. FT-IR data analysis on amide I band was performed for the sample BGI2 and lyophilized BG. The band was fitted by Gaussian functions after a six point Savitsky–Golay smoothing by means of Origin software. The number and initial positions of the Gaussian peaks were obtained by the second derivative spectra. An initial half-bandwidth of 7 cm^−1^ was automatically generated by the software.

Transmission electron microscopy (TEM) was performed with a PHILIPS EM208S microscope equipped with a Mega View camera for digital acquisition of images. The textural properties of WSNs (pore diameters, pore volume, and BET surface area) were already determined [33].

### 2.5. Catalytic Assays

The activity of free BG and immobilized BGI2 and BGI9 was determined at 50 °C using a solution 8 mM of cellobiose in buffer at pH 5.0. The amount of BG was 0.15 mg/mL for free and immobilized BGs. At fixed times, 2 mL of the solution was withdrawn and heated at 100 °C for 10 min to inactivate the enzyme. To verify that we did not artificially generate glucose by thermal hydrolysis, we analyzed a blank (cellobiose in water without the enzyme in standard test conditions) and found that there was no glucose in the blank after the thermal treatment at 100 °C. The aliquots containing the immobilized BG were centrifuged for 7 min at 11,000 rpm. Glucose (GO) assay kit was used for measuring glucose concentration. The amount of released glucose was determined by incubating an appropriate amount of quenched reaction mixture (1 mL), previously diluted, with 2 mL of glucose-measuring reagent at 37 °C for 30 min, on the basis of the D-glucose oxidase–peroxidase method. In particular, glucose is oxidized to gluconic acid and hydrogen peroxide by glucose oxidase. Hydrogen peroxide reacts with *o*-dianisidine in the presence of peroxidase to form a colored product (oxidized *o*-dianisidine), which reacts with sulfuric acid to form a more stable colored product. The absorbance (OD) measured at 540 nm by a spectrometer (Perkin Elmer Instruments, Lambda 25 UV-VIS) is proportional to the original glucose concentration [38]. Each experiment was performed in triplicate.

The kinetic parameters were evaluated by measuring initial reaction rates at varying substrate concentrations (from 3 to 10 mM) within the zone of linear relationship between product concentration and time. The obtained data were linearized by Lineweaver–Burk plot [39].

$$\frac{1}{v}=\frac{K_{M}}{V_{\max}}\cdot \frac{1}{s}+\frac{1}{V_{\max}}$$

where ***v*** is the reaction rate in μmol/L∙min and ***s*** is the substrate concentration in mmol/L. Plotting the reciprocal of initial reaction rate versus initial substrate concentration, ***V_max_*** and ***K_M_*** can be easily determined from the *x*-axis (−1/***K_M_***) and *y*-axis (1/***V_max_***) intercepts.

The activity of free BG was also measured in acetone. Free β-glucosidase activity was determined by measuring the amount of glucose obtained after 20 min of reaction using as solvent anhydrous acetone. The test was conducted using cellobiose 4.7 mM, anhydrous acetone and 0.15 mg/mL of BG at 40 °C. The enzyme was inactivated at 100 °C for 10 min.

### 2.6. Thermal Stability

The optimum temperature for the free and immobilized enzyme activity was evaluated by incubating the enzyme solution for 1 h at a certain temperature (50, 60, 70, and 80 °C) at pH 5 and then adding cellobiose (8 mM). The reaction was stopped after 1 h by keeping the solution at 100 °C for 1 h. Finally, the glucose concentration in the reaction batch was determined.

### 2.7. Operational Stability

The immobilized samples BGI2 and BGI9 were analyzed in consecutive reaction cycles to assess their reusability. Each reaction was carried out at 50 °C and pH 5.0. After each reaction cycle, the solution was analyzed to determine glucose concentration and the biocatalyst powders were separated by centrifugation, washed with the buffer solution, and reused. The hydrolysis yield (mole of glucose/2 × initial mole of cellobiose) after the first cycle was used as the control.

## 3. Results and Discussion

The methodology used to immobilize the enzyme is based on the principles of supported homogeneous catalysis—the active species are anchored on a solid matrix through a linker of suitable length. This approach preserves the quality of both homogeneous and heterogeneous catalysis, that is, high activity is combined with ease of separation of the catalyst. Furthermore, the presence of a flexible arm can decrease the possible steric hindrance of the enzyme promoted by the chemical linkage [40].

**Scheme 1:**
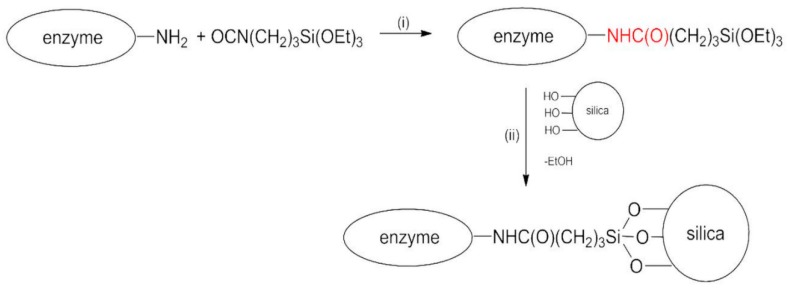
Reaction scheme for covalent immobilization of BG.

Taking advantage of previous studies [41,42], the choice of the spacer was suggested by the simultaneous presence of –NH_2_ groups on the enzyme and –OH groups on the silica nanoparticles. In fact, the primary structure of all proteins is rich in –NH_2_. For the same reason, one well-known crosslinking agent conventionally used in enzyme covalent immobilization is glutaraldehyde [43]. On this basis, the synthetic strategy described by Scheme 1 was followed.

The whole procedure is carried out in anhydrous acetone under nitrogen flux.

In step (i), the reaction between the enzyme and the bifunctional molecule TEPI in anhydrous acetone occurred, with the formation of a steady urethane bond (in red) between the reactants. Subsequently (step (ii)), silica nanoparticles were added in situ, and the catalyst was anchored to the solid support via hydrolysis of the ethoxysilyl groups. Bradford analysis was performed on the supernatant obtained after the first centrifugation of the samples in acetone. It indicated a complete absence of the protein, this being the protein insoluble in acetone. The same analysis conducted on the first washing buffer solution showed an immobilization yield of 90% and 50% for BGI9 and BGI2, respectively. This means that almost all the enzyme introduced in the reaction system was bound to the support using 9 μL of TEPI, whereas only half was bound by using 2 μL of TEPI. The immobilization efficiency was 0.15 and 1.2 for BGI9 and BGI2, respectively. The amount of immobilized enzyme was 27% and 15% for BGI9 and BGI2, respectively.

To determine whether acetone can inactivate the enzyme, free BG activity was measured in anhydrous acetone, as specified in the Materials and Methods section. The results indicated that BG was hyperactive in acetone, converting 93.8% wt of cellobiose in glucose in only 20 min. The same conversion values were obtained for free BG in water at 150 min and for BGI2 in water at 120 min.

**Figure 1:**
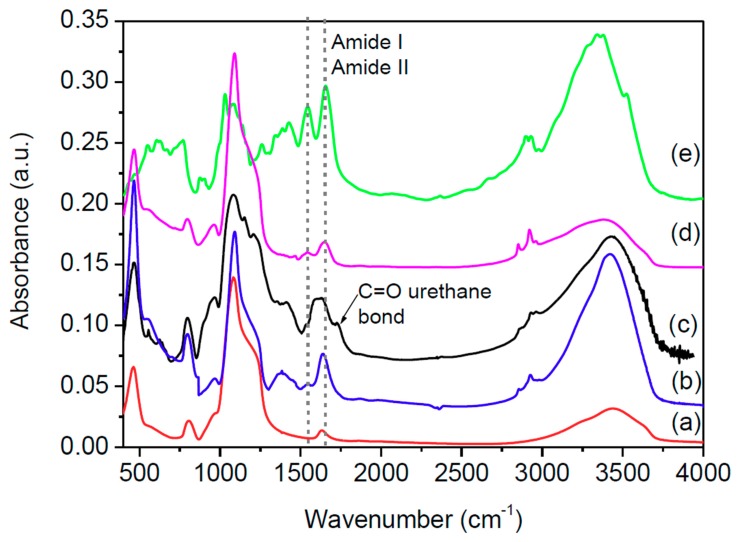
FT-IR spectra of silica nanoparticles (**a**) BGI2, (**b**) BGI9, (**c**) β-glucosidase (BG) on wrinkled silica nanoparticles (WSNs) without triethoxysilylpropylisocyanate (TEPI) (**d**) and lyophilized BG (**e**).

The FT-IR spectra of the bio-conjugate compounds, WSNs alone, and lyophilized BG are displayed in Figure 1. Here, we also report the spectrum of BG adsorbed on WSNs in the absence of TEPI, that is, by physical adsorption. The presence of the BG was testified by the bands between 1500 and 1700 cm^−1^, comprising amide I and amide II bands of the polypeptide backbone [44]. The absence of a band at 2270 cm^−1^, attributed to the stretching of the reactive group –N=C=O of TEPI [45], was indicative of the absence of unreacted TEPI. The formation of the urethane bond was evidenced by the appearance of a peak at 1720 cm^−1^, assigned to the hydrogen bonded urethane carbonyl [46]. The urethane band was visible in the spectrum of BGI9 because there was both more TEPI and more BG. It was barely visible in the spectrum of BGI2 because the amount of these links was much smaller.

From the FT-IR spectra, it was shown that BGI9 underwent a strong conformational modification. Amide I and amide II in the spectrum of BGI9 were quite superimposed due to a shift of amide I band towards lower wavenumbers, indicating a high degree of unfolding/aggregation [47]. Instead, BGI2 had amide I and amide II bands in about the same positions and intensity ratio as lyophilized BG, pointing out a smaller modification of the protein conformation. The same was observed for BG physically immobilized on silica in the absence of TEPI. Bands related to Si−O−Si vibration modes appeared around 460, 800, and 1080 cm^−1^. The positions of these bands were indicative of a dense silica network [23,24].

**Figure 2:**
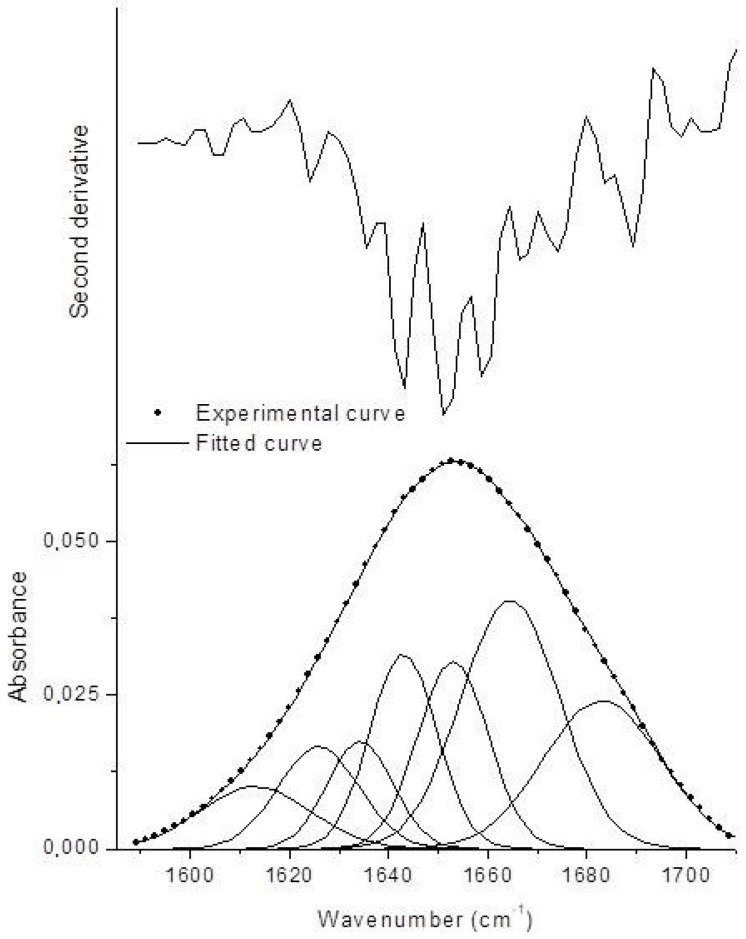
Second derivative spectrum and fitted amide I curve of BGI2 (χ^2^ = 1.27 × 10^−7^, *R*^2^ = 0.99985).

To determine the degree of modification of the polypeptide conformation, amide band I fitting was carried out, as specified in the Materials and Methods section. Amide I band originated mainly from the C=O stretching vibration of the peptide group, whose frequency was influenced by the strength of hydrogen bonds and the dipole–dipole interactions between carboxyl groups along the chain. This band consists of overlapping components representing secondary structure elements [47]. The results of amide I band fitting are reported in Figure 2.

The Gaussian components in the range 1620–1640 cm^−1^ were associated with β-sheets, in the range 1640–1650 cm^−1^ with random coils, in the range 1650–1660 cm^−1^ with α-helices, and 1650–1660 cm^−1^ with β-turns. Gaussian components at lower and higher wavenumbers are indicative of protein aggregation through intermolecular hydrogen bonding [48]. The amount of the different secondary structure elements was estimated by the ratio between the area underneath each Gaussian component and the total area underneath the amide I band. The results are reported in Table 1 and compared with those of lyophilized BG. Upon immobilization, BG underwent some conformational changes concerning an increase of β-turns and aggregates at expenses of α-helices. Random coil (disordered) structure did not increase, meaning that the immobilized BG retained an ordered structure. These results are coherent to what has been reported in the literature for BG immobilized on smectite nanoclays, where the same kinds of modifications were found but the enzyme retained its catalytic activity [48]. The difference is that in our case β-sheets were less influenced.

A structural characterization of the bio-conjugate catalysts was performed. The whole immobilization process can be considered as occurring in three steps. The first two have already been illustrated; the final one was the removal of the bio-conjugated catalyst from the reaction environment. It was then necessary to understand in which one the modification of the native structure of the enzyme took place.

**Figure 3:**
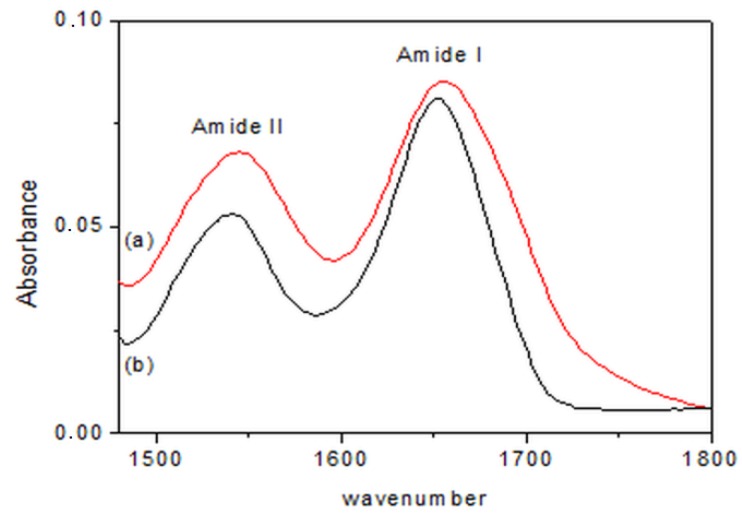
FT-IR spectra of (**a**) lyophilized BG and (**b**) BG after the first step of reaction.

In Figure 3, representative FT-IR spectra of the products obtained from step (i) and of lyophilized BG are compared. The spectra of this intermediate product were the same for both preparations. The spectra are displayed in the 1500–1800 cm^−1^ range. With the exception of a very slight displacement of the amide I band, the two bands were substantially unchanged—the native structure of the protein was slightly modified at this stage, although it involved the direct chemical attack on the enzyme. Thus, the removal of the bio-conjugated catalyst from the reaction environment seemed to be the critical step influencing the polypeptide conformation.

**Figure 4:**
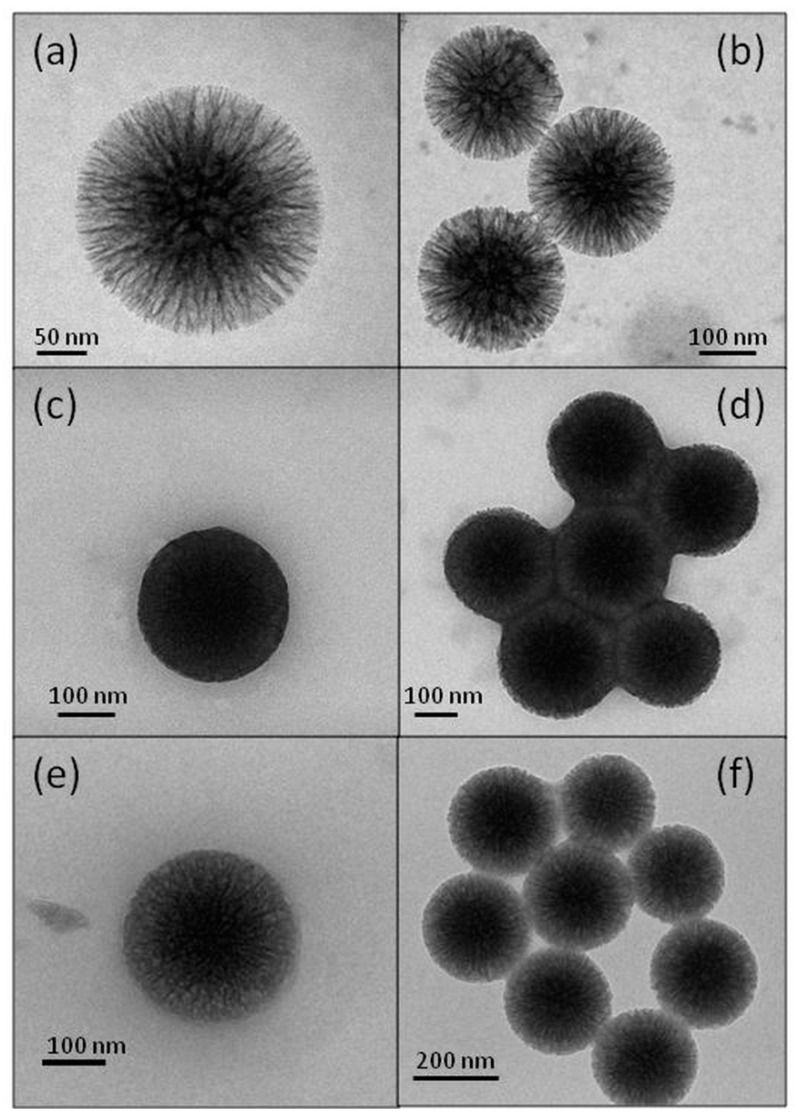
Transmission electron microscopy (TEM) micrographs of WSN (**a**,**b**), BGI9 (**c**,**d**), and BGI2 (**e**,**f**).

TEM micrographs of the pristine and bio-conjugated wrinkled silica nanoparticles are displayed in Figure 4. For BGI9, the nanoparticle seemed nearly saturated with the enzyme (Figure 4c), and there was a strong sticking tendency between the nanoparticles (Figure 4d). The images of BGI2 showed a smaller filling degree (Figure 4e), confirming the Bradford result, and an apparently smaller sticking tendency (Figure 4f). These results also confirmed the Bradford analysis for determining the immobilization yield. They suggest that in BGI9, the BG molecules were functionalized in many positions. We hypothesized that the enzyme molecules anchored to the external surface acted as a bridge between several nanoparticles. Silica nanoparticles are strongly hydrophilic and tend to disperse in water. When the biocatalyst was placed in water and the enzyme acquired conformational mobility, the stresses exerted by the bridged nanoparticles caused its denaturing, as evidenced by the FT-IR analysis. This behavior did not occur in the BGI2, where the TEPI local concentration was smaller. Furthermore, in BGI2, there was less crowding of the protein in a confined space as a possible cause of denaturation.

The kinetics of cellobiose hydrolysis was studied recording the product concentration versus time of the reactions catalyzed by free and immobilized BGs. Both the immobilized enzymes and the free enzyme followed a Michaelis−Menten behavior. Lineweaver−Burk plots of the experimental data allowed calculating the kinetic constants reported in Table 2.

The data are the mean value with standard deviation from triplicate experiments. The reaction conditions were pH 5.0 (citrate buffer 8 mM), T of 50 °C.

The sample BGI2 had about the same ***V_max_*** of the free enzyme, as expected, as the structural analysis evidenced that the enzyme retained its ordered structure. The apparent ***K_M_*** was almost half of that of the free enzyme. In the literature, an increase in the ***K_M_*** value was often observed upon immobilization [49,50,51,52,53]. Only in some instances, a decrease of ***K_M_*** following immobilization has been reported [54,55]. A smaller apparent ***K_M_*** indicated that the immobilized enzyme had higher affinity for its substrate or that there was an enhanced substrate concentration near the active sites caused by the interactions between the substrate and the matrix. Actually, WSNs contain a great number of surface silanol Si–OH, as testified by the presence of a band 970 cm^−1^ in the FT-IR spectra of Figure 1 (stretching vibration of Si–OH) [56]. Surface silanols can interact with cellobiose via hydrogen bonding, causing an increased local concentration [57]. The apparent ***K_M_*** for the BGI9 biocatalyst was about twice that of free BG, whereas ***V_max_*** was about half. The increase in the apparent ***K_M_*** upon immobilization suggested mass transfer limitations, due to both the sticking of the nanoparticles and a crowding effect due to the excessive amount of enzyme inside the pores. The decrease in ***V_max_*** may be ascribed to detrimental modifications in the enzyme conformation for the bridging enzymes [50] and a loss of the conformational flexibility caused by interactions between the polypeptide molecules and with the matrix [58]. Concerning the immobilization of BG on nanoparticle carriers, Verma et al. [27] immobilized a thermostable enzyme (β-glucosidase from *Aspergillus niger*) on functionalized magnetic nanoparticles by covalent binding. These biocatalysts showed a ***K_M_*** value of 3.5 and 4.3 mM for free and immobilized BG, respectively. Zheng et al. [28] immobilized BG on magnetic chitosan microspheres and found a small increase in ***K_M_*** for the immobilized enzyme (6.46 vs. 4.94 mM). Also, Singh et al. [20] found an increase in ***K_M_*** (3.8 vs. 2.5 mM) for BG covalently immobilized onto functionalized silicon oxide nanoparticles. On the other hand, Agrawal [29] et al. immobilized BG onto Stober silica nanoparticles through glutaraldehyde crosslinking, finding a smaller increase in ***K_M_***, from 0.9 to 1.1 mM. The decrease in ***K_M_*** value found in our case can be attributed to both the peculiar morphology of WSNs and the presence of the flexible spacer. Both contributed to the reduction of diffusion limitations. WSNs have a central-radial pore structure that widen radially outward, enhancing the accessibility of the substrate to the enzyme, and their high surface area maximizes the surface silanol amount for interaction through hydrogen bonding with the substrate. Actually, we also found a decrease in ***K_M_*** for BG physically adsorbed onto WSNs [33], but the decrease was smaller than that in the present study (4.3 vs. 2.5 mM).

**Figure 5:**
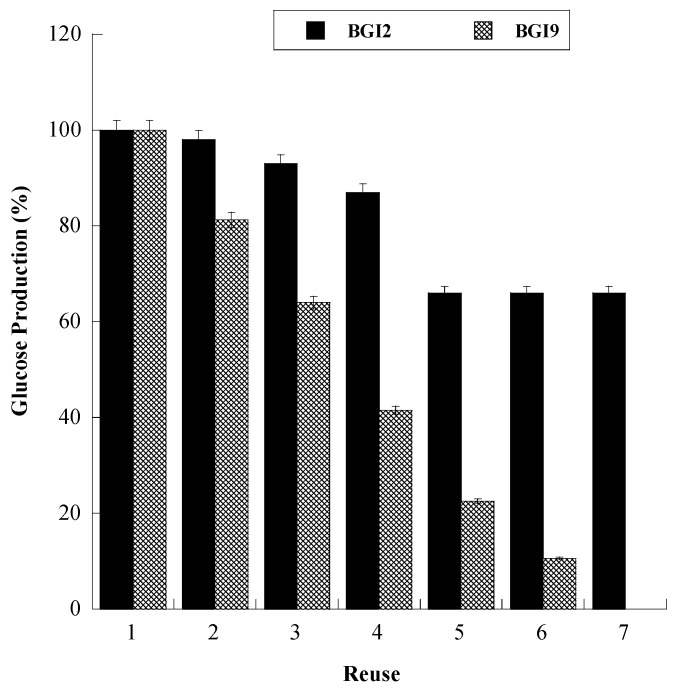
Operation stability of immobilized BG during cellobiose conversion for BGI2 (full bars) and BGI9 (empty bars). The data are the mean value with standard deviation from triplicate experiments.

The immobilized BGs were used repeatedly in six consecutive 24 h hydrolysis cycles. The reusability of immobilized enzymes is a very important characteristic for large-scale applications. The results are displayed in Figure 5. In both cases, the enzyme demonstrated operational stability, as it could be reused for several times. However, BGI2 had much better performance. In fact, BGI2 was reused for seven times, holding 70% of its activity, whereas BGI9 preserved only 10% of its initial activity after the sixth reuse. Because in both cases the enzyme was covalently bound, we did not attribute this behavior to leaching. For BGI9, a macroscopic phenomenon of aggregation was observed during reuse, leading to eye-visible particles, which could be responsible for enzyme inactivation or inaccessibility of the enzyme to the substrate. In the case of BGI2, this phenomenon was not observed. For BGI2, a contribution to the decrease of the reaction yield could have been due to the physical loss of small amounts of biocatalyst during the transfer procedures.

Compared with other BG/nanoparticle bioconjugates, Singh et al. [20] found that after the 25th cycle, the immobilized BG showed up to 95% residual activity. Verma et al. [27] found that immobilized BG retained residual activity higher than 80% up to the eighth reuse. Zheng et al. [28] found that 84.4% of the original BG activity was recovered after repeated hydrolysis of eight batches. In these last two cases, the magnetic particles greatly facilitated the separation of the immobilized BG. Agrawal et al. [29] found instead that BG kept 70% of its original activity after the 10th cycle of reuse, which is analogous to our results.

The effect of temperature on the catalytic activity was evaluated for the best performing sample.

**Figure 6:**
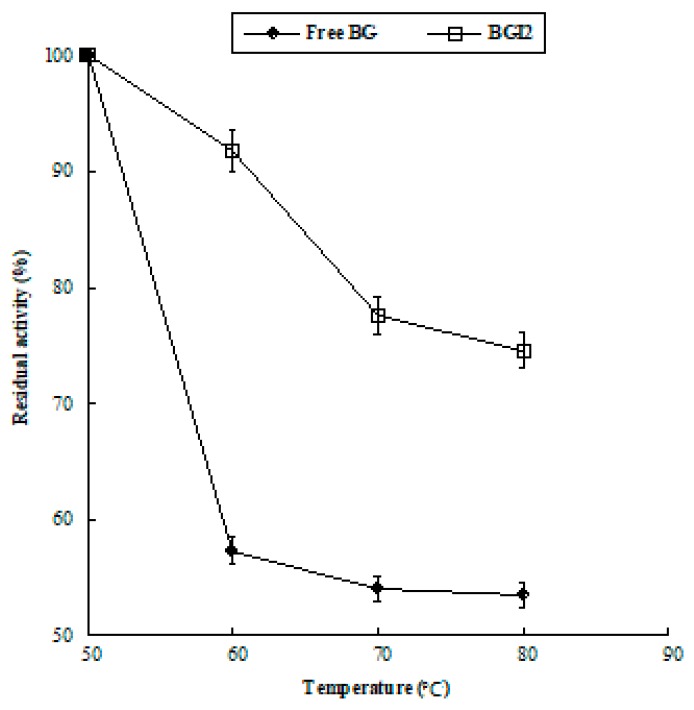
Residual activity (%) at different temperatures. The data are the mean value with standard deviation from triplicate experiments.

In Figure 6, the percentage residual activities at different temperatures are displayed for free BG and BGI2. The immobilized enzyme showed increased thermal stability with respect to the free form. The free enzyme lost 43% of its percentage activity after incubation at 60 °C for 1 h and about 47% after incubation at 80 °C for 1 h. On the contrary, the immobilized enzyme retained 74.6% of its percentage activity after incubation at 80 °C for 1 h. An improvement of thermal stability of β-glucosidase upon immobilization has been often observed [50,51,52]. It was attributed to the formation of multipoint attachment to the support that inhibited the conformational freedom and thermal vibration, inducing an increase in enzyme rigidity that prevents the immobilized protein from unfolding and denaturing under high temperature conditions [59,60].

## 4. Conclusions

An enzyme immobilization protocol was developed and optimized through covalent binding of β-glucosidase into wrinkled silica nanoparticles. The immobilization reaction exploited a bifunctional linker of suitable length and it was based on the use of an organic solvent (anhydrous acetone) in which the enzyme was insoluble. Two different amounts of the crosslinking agent were chosen. The linker/enzyme molar ratio seemed to be the key factor that affected the performance of biocatalyst. The best performing biocatalyst obtained showed increased catalytic performance through a decrease of the apparent ***K_M_***. A smaller apparent ***K_M_*** indicated that a very favorable micro-environment was created in the interior of the nanopores, improving the enzyme catalytic activity.

The biocatalyst showed a very good operational stability and thermal stability. This procedure, based on the formation of a urethane bond between the crosslinker and the polypeptide, could be applied to other enzymes because the primary structure of proteins is rich in –NH_2_ groups.

## Figures and Tables

**Table 1:** Percentage of secondary structure elements for BGI2 and lyophilized BG.

Structure	Lyophilized BG	BGI2
α-Helices	28.9	14.6
β-Sheets	13.9	17
β-Turns	35.4	46.4
Unordered	16.4	14.4
Aggregates	5.4	7.6

**Table 2:** Kinetic constants of free and immobilized BG.

Enzyme	*K_M_* (mM)	*V_max_* (μmol/min∙mg)
Free	4.74 ± 0.03	26.2 ± 0.20
BGI9	10.90 ± 0.03	13.3 ± 0.01
BGI2	2.50 ± 0.01	25.0 ± 0.02
